# Valorization of lignocellulosic wastes for sustainable xylanase production from locally isolated *Bacillus subtilis* exploited for xylooligosaccharides’ production with potential antimicrobial activity

**DOI:** 10.1007/s00203-023-03645-2

**Published:** 2023-08-21

**Authors:** Hamada El-Gendi, Ahmed S. Badawy, Elsayed K. Bakhiet, Mohammed Rawway, Salah G. Ali

**Affiliations:** 1 Bioprocess Development Department, Genetic Engineering and Biotechnology Research Institute, City of Scientific Research and Technological Applications (SRTA-City), New Borg El-Arab City, Alexandria, 21934 Egypt; 2 Botany and Microbiology Department, Faculty of Science, AL-Azhar University, Assiut, Egypt

**Keywords:** Xylanase, Lignocellulosic wastes, Xylooligosaccharides, Multidrug-resistant

## Abstract

The worldwide availability of lignocellulosic wastes represents a serious environmental challenge with potential opportunities. Xylanases are crucial in lignocellulosic bio-hydrolysis, but the low enzyme productivity and stability are still challenges. In the current study, *Bacillus subtilis* (coded ARSE2) revealed potent xylanase activity among other local isolates. The enzyme production optimization revealed that maximum enzyme production (490.58 U/mL) was achieved with 1% xylan, 1.4% peptone, and 5% NaCl at 30 °C and pH 9. Furthermore, several lignocellulosic wastes were exploited for sustainable xylanase production, where sugarcane bagasse (16%) under solid-state fermentation and woody sawdust (2%) under submerged fermentation supported the maximum enzyme titer of about 472.03 and 485.7 U/mL, respectively. The partially purified enzyme revealed two protein bands at 42 and 30 kDa. The partially purified enzyme revealed remarkable enzyme activity and stability at 50–60 °C and pH 8–9. The enzyme also revealed significant stability toward tween-80, urea, DTT, and EDTA with *V*_*max*_ and *K*_*m*_ values of 1481.5 U/mL and 0.187 mM, respectively. Additionally, the purified xylanase was applied for xylooligosaccharides production, which revealed significant antimicrobial activity toward *Staphylococcus aureus* with lower activity against *Escherichia coli*. Hence, the locally isolated *Bacillus subtilis* ARSE2 could fulfill the xylanase production requirements in terms of economic production at a high titer with promising enzyme characteristics. Additionally, the resultant xylooligosaccharides revealed a promising antimicrobial potential, which paves the way for other medical applications.

## Introduction

The continuous accumulation of lignocellulosic wastes represents a great growing environmental challenge. Cellulose and hemicellulose are the major constituents of agricultural wastes that could be suitable candidates for sustainable biorefinery industries (Devi et al. 2022). Several attempts were directed at the valorization of such substrates; however, high cost invested and low selectivity remain challenging (Malhotra and Chapadgaonkar 2018; Trejo et al. 2022). From the environmental perspective, the application of enzymes for lignocellulose hydrolysis gained much attention as an eco-friendly and cost-effective alternative for biomass conversion (Sunkar et al. 2020). Xylan represents 20–30% of hemicellulose, and hence, xylanases were widely reported as a promising tool for efficient agricultural wastes valorization to value-added products (Sunkar et al. 2020; Ontañon et al. 2021). Xylanases catalyze the hydrolyze β-1,4 glycosidic linkages in xylan to release β-d-xylopyranosyl mono-, di-, and trisaccharides (Malhotra and Chapadgaonkar 2018). The resulting saccharides are amenable to microbial fermentation which opens the scope for diverse promising applications (Ghosh et al. 2021; Vasić et al. 2021). Currently, xylanases are ranked among the most demanded enzymes with a large market share attributed to their numerous industrial applications (Van Hoeck et al. 2021; Golgeri et al. 2022).

Xylanases have recently acquired industry interest in biofuel production, pharmaceutical sectors, specific chemical manufacturing, wood pulp bioleaching for papermaking, food/beverage manufacturing, and animal nutrition (Basit et al. 2020; Qeshmi et al. 2020; Ghosh et al. 2021; Miao et al. 2021). The growing demand for xylanases forced their large-scale production at a cost-effective and commercial-scale level (Ramanjaneyulu and Rajasekhar Reddy 2016). Among others, microbial xylanases fulfill the biotechnological requirements for large-scale production in terms of scalability and production titer (Basit et al. 2020). Different microbial sources were reported for xylanase production including yeasts (Miao et al. 2021; Šuchová et al. 2022), fungi (Cekmecelioglu and Demirci 2020; Patel and Dudhagara 2020a), and actinomycetes (Danso et al. 2022). Currently, eubacteria are largely applied at commercial-scale xylanases’ production (Chakdar et al. 2016; Ghosh et al. 2021). Several bacterial genera are reported for xylanases production, including *Cellulomonas, Paenibacillus,* and *Micrococcus* with dominant superiority of* Bacillus* species in terms of production efficiency and enzyme characteristics (Mmango-Kaseke et al. 2016; Patel and Dudhagara 2020b; Ghosh et al. 2021; Ontañon et al. 2021). Regarding the high production cost using pure xylan, sustainable xylanase production is currently directed toward the utilization of agricultural wastes along with the development of efficient production strategies (Biswas et al. 2010). Hence, several agricultural wastes and food industries were implied in the economic xylanase production process such as wheat straw, rice straw, and sugarcane bagasse. This direction not only reduce the production cost, but also reduce the environmental impacts of such wastes.

Furthermore, the xylooligosaccharides (XOS) attract great intention regarding their promising biological activities including anticancer, anti-inflammatory, and antioxidant characteristics (Gowdhaman et al. 2014; Ghosh et al. 2021). Regarding the widespread of multidrug-resistant microbial pathogen, a few studies reported the antimicrobial potentials of XOS against some human pathogens. Therefore, it was of interest to explore xylanases production using local bacterial isolate and optimize the enzyme production process on low-cost agricultural and industrial residues. Furthermore, the enzyme characteristics and kinetics was evaluated for purified enzyme. Additionally, the XOS resulted from xylan hydrolysis with purified enzyme were evaluated for potential antimicrobial activity against some multidrug-resistant pathogens.

## Materials and methods

### Bacterial isolation and purification

For the isolation of xylan-degrading bacteria, six samples were collected from three different sources (Assiut, Egypt), including garden soil (27°12′16.42″N, 31°10′48.06″E), decayed agricultural wastes (27°11′39.71″N, 31°31′45.53″E), and ruminant dung (27°12′16.06″N, 31°6′51.46″E). All samples (10 g/sample) were serially diluted (10^–1^–10^–6^) in sterile saline solution (0.9% NaCl) under shaking for 1 h. Under aseptic conditions, 100 µL from the serially diluted samples was separately spread into xylan agar (XA) medium containing (g/L): xylan, 5.0; peptone, 5.0; yeast extract, 5.0; K_2_HPO_4_, 1.0; MgSO_4_.7H_2_O, 0.2; and agar 15, at pH 7.0. All plates were incubated at 37 °C for 72 h. The growing separate colonies were picked out and streaked again on XA plates to ensure purity.

### Screening of xylanase activity on XA plates

All purified bacterial isolates were separately streaked on the XA plates and incubated at 37 °C for 72 h. The xylanolytic activity was assessed by flooding the plates for 15 min with 0.1% (w/v) Congo red (Sigma-Aldrich, USA). Afterward, the stain was discarded, and the excess stain was removed with 1 M NaCl for 10 min. The developing clear zones (Halo-zones) around bacterial colonies indicate xylanase activity (Samanta et al. 2011).

### Quantitative determination of xylanase activity

The xylanase activity was further evaluated quantitatively through the dinitrosalicylic acid method (DNS) using birchwood xylan (Sigma-Aldrich, USA) as a substrate (Bailey et al. 1992)**.** First, all bacterial isolates with xylanase activity were inoculated separately (5% inoculum, 1.0 × 10^5^ CFU/mL) into 100 mL of xylanase broth medium (XB) and incubated under shaking (200 rpm) at 37 °C for 72 h. Afterward, the cell-free supernatants (CFS) were used as a source for crude enzymes. The CFS (0.2 mL) was added to 1.8 mL of birchwood xylan (1.0% prepared in 0.05 M sodium citrate buffer, pH 5.3) and incubated at 50 °C for 10 min. The reaction was stopped by 3.0 mL of DNS solution and then boiled at 100 °C for 15 min**.** After cooling, the developed color was measured at 540 nm. The enzyme activity (one unit) is defined as the amount of enzyme required to release 1 μmol of xylose/min under the defined assay conditions.

### Identification of the potent xylanase-producing isolate

The most potent xylanase-producing isolate was identified according to morphological and biochemical characteristics. Furthermore, sugar fermentation and substrate assimilation, including starch, gelatin, carboxymethyl cellulose (CMC), etc., were also evaluated.

At the molecular level, the isolate was identified according to the 16S rRNA sequencing approach. The total genomic DNA was extracted using a DNA isolation kit (Axygen, Biosciences Co., USA). Later, two universal primers were used to amplify the 16S rRNA gene: 27F (5′-GAGAGTTTGATCCTGGCTCAG-3′) and 1492R (5′-TACCTTGTTACGACTT-3′) through polymerase chain reaction (PCR). The PCR product was purified through QI quick DNA purification kit (Qiagen, USA) according to the manufacturer’s instructions and sequenced by a 3130X-automated DNA sequencer (Applied Biosystems, USA). The 16S rRNA gene sequence of strain ARSE2 was compared with the reference sequences published on the National Center for Biotechnology Information GenBank (NCBI GenBank) database using the Basic Local Alignment Search Tool (BLAST). Moreover, the sequence alignment was carried out by ClastalW, and the phylogenetic tree was constructed and analyzed using MEGA 6 software.

### Optimization of xylanase production conditions

Various nutritional factors and cultivation conditions were evaluated through a one-variable-at-a time approach to maximize the xylanase production through ARSE2 isolate. Generally, the XB was used as a basal production medium and xylanase activity was measured after 48 h of incubation at 37 °C, under shaking conditions of 200 rpm (except for temperature and incubation time effects). Additionally, the bacterial growth was also determined at 600 nm (OD_600_).

#### Effect of different carbon sources on xylanase production

To determine the best carbon source for xylanase production, various simple and complex carbon sources, including glucose, lactose, fructose, sucrose, starch, CMC, mannose, xylose, ribose, galactose, and birchwood xylan, were used separately as the sole source of carbon (1% w/v).

#### Effect of different nitrogen sources on xylanase production

Various organic and inorganic nitrogen sources were screened for their ability to support maximal xylanase production. The organic nitrogen sources included gelatin, tryptone, peptone, yeast extract, and beef extract, while the inorganic nitrogen sources were ammonium sulfate (NH_4_)_2_SO_4_, ammonium nitrate (NH_4_NO_3_), sodium nitrate (NaNO_3_), ammonium dihydrogen phosphate (NH_4_H_2_PO_4_), and urea. Each nitrogen source was used at a final concentration of 0.5% (w/v) in the XB medium. Furthermore, the optimum level of the nitrogen source supporting the highest xylanase activity (optimum nitrogen source) was investigated at different concentrations in the range of 0.2–8% (w/v).

#### Effect of sodium chloride (NaCl) concentrations on xylanase production

The effect of NaCl on xylanase production was evaluated by supplementing the XB medium with different concentrations of NaCl from 1 to 7% (w/v).

#### Effect of different metals and amino acids on xylanase production

Different metals, including potassium chloride (KCl), calcium chloride (CaCl_2_), nickel chloride (NiCl_2_), barium chloride (BaCl_2_), cobalt chloride (CoCl_2_), magnesium chloride (MgCl_2_), and copper chloride (CuCl_2_), were investigated for xylanase production, at 0.05% final concentrations. Additionally, seven different amino acids were investigated for their effects on xylanase production, including lysine, glycine, cysteine, serine, methionine, tryptophan, and glutamic acid. Controls without any supplemented metals or amino acids were included.

#### Effect of cultivation temperature and medium pH on xylanase production

To determine the optimum temperature for xylanase production, the potent xylanase-producing isolate was cultivated at different incubation temperatures (20–60 °C) on the XB medium. On the other hand, to determine the optimum pH for xylanase production, the isolate was grown on XB at 37 °C with different initial pH values of 3.0–11.0. The enzyme activity was determined after 48 h of incubation at each specified temperature or pH value.

#### Effect of inoculum size and incubation period on xylanase production

Different inoculum sizes [1, 2, 3, 4, 5, 6, 8, 10, and 14% (v/v)] from the potent xylanase producers were inoculated in 250 mL Erlenmeyer flasks containing 100 mL of the sterilized XB medium. To prepare the inoculation pre-culture, the potent xylanase producer (ARSE2) was grown on nutrient broth for 18 h at 37 °C.

On the other hand, the optimum incubation period for maximum xylanase production was evaluated by following the enzyme production for 108 h on the XB medium and incubated at 37 °C under shaking (200 rpm). Samples were withdrawn under aseptic conditions every 12 h and assayed for both enzyme activity and bacterial growth (OD_600_).

#### Lignocellulosic waste types and pre-treatment

Xylanase production was evaluated using different agricultural and industrial wastes, including wheat straw, rice straw, sugarcane bagasse, corn stalks, woody sawdust, cotton stalks, and bean straw. The wastes were first air-dried and ground into a fine powder. Afterward, the powders were separately treated with NaOH solution (1%) in a final solid:liquid ratio of 1:10 for 2 h at room temperature. The treated powder was filtered to remove the excess base, washed several times with distilled water, and autoclaved (final substrate: distilled water ratio of 1:10) for 1 h. After that, the treated substrates were filtered and washed with distilled water until the wash water became neutral and then air-dried.

#### Application of treated lignocellulosic wastes for xylanase production under solid-state (SSF) and submerged fermentation (SF)

The effect of treated agro-industrial wastes on xylanase production was evaluated under SSF and SF conditions. In SSF, 16 g of each pre-treated substrate was added to a 250 mL Erlenmeyer flask containing 100 mL of the following mineral salt solution (g/L): MgSO_4_.7H_2_O, 0.3; K_2_HPO_4_, 2.0 at pH 7.0. After autoclaving for 15 min, all flasks were inoculated with an overnight culture of ARSE2 isolate and incubated at 37 °C for 72 h under static conditions. Afterward, xylanase enzymes were extracted with 100 mL of 50 mM sodium citrate buffer (pH 5.3) and filtered. The CFS was used as a source for xylanase activity. On the other hand, to study the effect of SF upon xylanase production, 2% of each waste was added separately to 250 mL Erlenmeyer flasks containing 100 mL of the previous mineral salt solution and incubated at 37 °C for 72 h under shaking (200 rpm).

### Partial purification of the produced xylanase

For enzyme purification, the most potent isolate (ARSE2) was incubated under optimum xylanase production conditions, and the CFS was used as a crude enzyme source. The protein contents of CFS were salted out by gradually adding ammonium sulfate up to 75% (w/v) saturation under mild stirring at 4 °C. After 24 h of stirring at 4 °C, the solution was centrifuged at 10,000 rpm for 15 min. The resulting protein pellet was then collected, dissolved in a minimum amount of phosphate buffer (50 mM, pH 7.1), and dialyzed (membrane cut off 10 KDa) against the same buffer for 48 h at 4 °C with four buffer changes. The dialyzed protein (2 mL) was then applied to a diethyl aminoethyl column (DEAE, HiPrep DEAE FF 16/10, Pharmacia, Sweden) pre-equilibrated with phosphate buffer (pH 7.1) through the GE AKTAprim plus system. The loaded protein was eluted with gradient NaCl solution (0–1000 mM) in phosphate buffer (pH 7.1) at a flow rate of 1 mL/min with a fraction size of 3 mL. The fractions revealing xylanase activity were collected, dialyzed, and concentrated through freeze drying. The xylanase activity and protein content in all fractions were evaluated and presented for each purification step. Furthermore, the purification progress was followed up through sodium dodecyl sulfate-polyacrylamide gel electrophoresis (SDS-PAGE) at 12% polyacrylamide gel as adapted from (Laemmli and Favre 1973).

#### Protein determination

Protein concentration was determined using direct measurement at a wavelength of 280 nm or according to the Folin-phenol reagent approach using bovine serum albumin (BSA) as the standard (Lowry et al. 1951).

### Characterization of the partially purified Xylanase

#### Effect of temperatures on activity and stability of xylanase

The impact of temperature on xylanase activity was measured by incubating the partially purified enzyme with xylan at different incubation temperatures in the range of 20–90 °C for 10 min. Furthermore, the enzyme thermal stability was evaluated by incubating the purified enzymes without substrate at temperatures 20–90 °C for 60 min. Then, the xylanase activity was measured as mentioned above and expressed as residual activity (%) considering activity at 60 °C without prior temperature treatment as control (100%).

#### Effect of different pH values on the activity and stability of xylanase

To evaluate the effect of different pH values on the activity of partially purified xylanase, the birchwood xylan was prepared separately in the following buffers (50 mM): citrate buffer (pH: 3.0, 4.0, 5.0, and 6.0); sodium phosphate buffer (pH: 7.0 and 8.0); glycine–NaOH buffer (pH: 9.0, 10.0, 11.0, and 12.0). The xylanase activity was determined at each pH value as mentioned above. The xylanase stability at different pH values (3.0–12.0) was determined by pre-incubating the enzymes at these pH values for 1 h prior to activity determination. Afterward, the xylanase activity was measured as mentioned above and expressed as residual activity (%), considering activity without prior pH incubation as control (100%).

#### Effect of different metal ions on the activity and stability of xylanase

The effect of some metal ions, including Ba^2+^, Mg^2+^, Ca^2+^, Ni^2+^, Co^2+^, and Mn^2+^ cations with different concentrations, including (1.0, 5.0, and 10.0 mM), was determined on the partially purified xylanase activity compared with control without any additions (100% activity). In addition, the enzyme stability against the previous metal ions at different concentrations was evaluated by pre-incubating the enzymes with each metal ion for 60 min before activity determination. The enzyme activity was expressed as a residual activity (%) compared to the control (without additions), which is considered 100% activity.

#### Effect of different detergents and enzyme inhibitors on the activity and stability of xylanase

Xylanase activity was determined in the presence of some detergents (Tween-80, Urea, and H_2_O_2_) at different concentrations (1, 5, and 10%). Additionally, the effect of some enzyme inhibitors such as ethylenediaminetetraacetic acid disodium salt (EDTA-Na_2_), SDS, and dithiothreitol (DTT) on the activity of the partially purified enzymes was determined (at different concentrations 1.0–10.0 mM) and compared with the control (100% activity). The stability of xylanase against the same detergents and enzyme inhibitors was measured by pre-incubating the enzymes with each detergent or enzyme inhibitor for 60 min. The enzyme activity was expressed as a residual activity (%) compared to the control (without any additives) as 100% activity.

#### Effect of substrate concentration on xylanase activity and kinetics

The effect of substrate concentration upon enzyme activity was evaluated by measuring the xylanase activity at different concentrations of birchwood xylan (0.5–5%) at pH 8 (sodium phosphate buffer). Lineweaver–Burk plots were applied to deduct the reaction kinetic constants: Michaelis–Menten constant (*Km*) and maximum enzyme velocity (*V*_*max*_) according to (Lineweaver et al. 1934).

### Evaluation of the antimicrobial activity of XOS

The antimicrobial activity of the prepared XOS was evaluated through the agar-well diffusion method against three human pathogens (Hudzicki 2009). The three applied pathogens: *Staphylococcus aureus* ATCC 25923 (*S. aureus*) as a Gram-positive bacterium, *Escherichia coli* ATCC 25922 (*E. coli*) as a Gram-negative bacterium, and *Candida albicans* ATCC 10231 (*C. albicans*) as a model for unicellular fungi were cultivated on Muller-Hinton broth for 24 h at 37 °C. The XOS samples were prepared by incubating birchwood xylan (1%, pH 7.1) with 40 µL of purified xylanase at 50 °C for different times: 15, 30, and 60 min. The agar-well diffusion was conducted through the separate distribution of 100 µL (0.5 McFarland) of each pathogen on Muller-Hinton agar plates. Under aseptic conditions, several wells (9 mm) were conducted on the agar surface and inoculated with 100 µL from each hydrolysis time. Non-hydrolyzed xylan and purified enzymes (100 µL) were also inoculated on each plate as negative controls. Additionally, two antimicrobial drugs, namely amoxicillin (AX-25, 25 mg/disk) and amphotericin-B (Am-B, 100 U/disk), were also included in the plates as positive controls for bacteria and fungi, respectively. All plates were incubated for 24 h at 37 °C, where the developed halo-zones around the wells indicated antimicrobial activity.

### Statistical analysis

The data included thought the current study are means (M) of three replicas with standard deviation (SD) from means represented as column with error bar in histograms or M ± SD in tables. Additionally, the cell size (length and width) of the potent xylanase-producing isolate was retrieved from the scanning electron microscopy (SEM) images through ImageJ software.

## Results and discussion

### Isolation and screening for xylanase activity

**Fig. 1:**
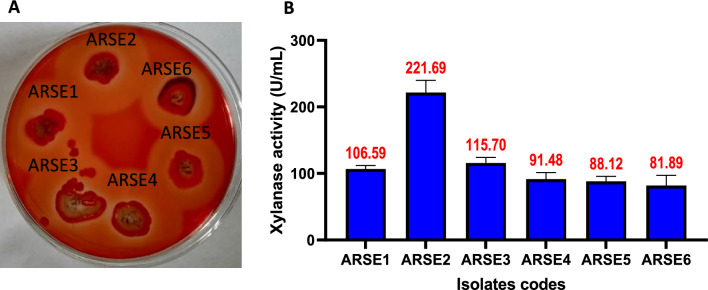
Qualitative screening of xylanase production on xylan agar plate (**A**) and the corresponding quantitative assay (**B**)

In the course of xylanase production screening, four serially diluted samples were inoculated separately on XA plates. Ninety-five separate bacterial colonies were detected on the XA plate’s surfaces, with about six colonies revealing clear zones (Halo-zones) around the bacterial growth (Fig. 1A).

Furthermore, the xylanase production potential of the six bacterial isolates (ARSE1-ARSE6) revealing halo-zones on XA plates was confirmed through quantitative xylanase determination. The results (Fig. 1B) asserted xylanase production ability for all examined bacterial isolates with varied production titers. Isolate coded ARSE6 reported the lowest xylanase production about 82 U/mL, where the most potent xylanase producer was isolate coded ARSE2 (221.69 U/mL). This variation in the production titer indicated the importance of screening and strain selection for enzyme production improving. Based on the quantitative xylanase determination, isolate ARSE2 was selected as most potent for the optimization of xylanase production and further studies.

**Fig. 2:**
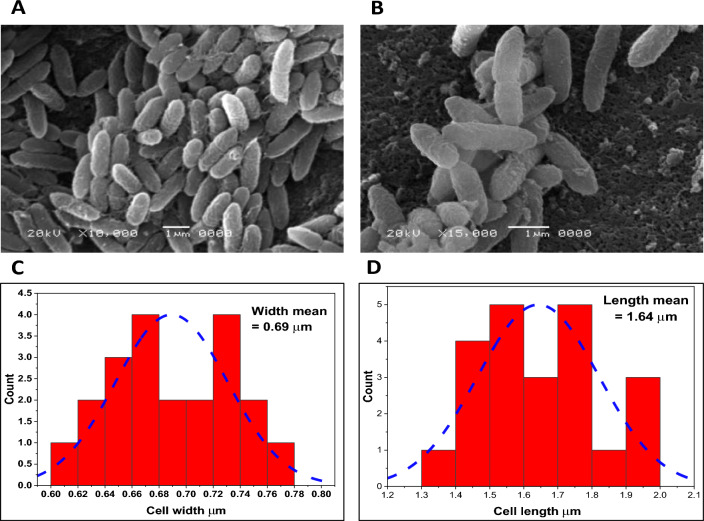
Scanning electron microscopy (SEM) for locally isolated ARSE2 with potent xylanase activity at 10000× (**A**) and 15000× (**B**) with cell dimensions’ measurements including cell width (**C**), and cell length (**D**) as deducted through Image J software

Several phenotypic characteristics for the potent xylanase-producing isolate (ARSE2) were evaluated. Under electron microscopy, the bacterium was diplobacilli (Fig. 2A, B) with average dimensions of 1.64 and 0.69 µm for cell length and width, respectively (Fig. 2C, D).

**Fig. 3:**
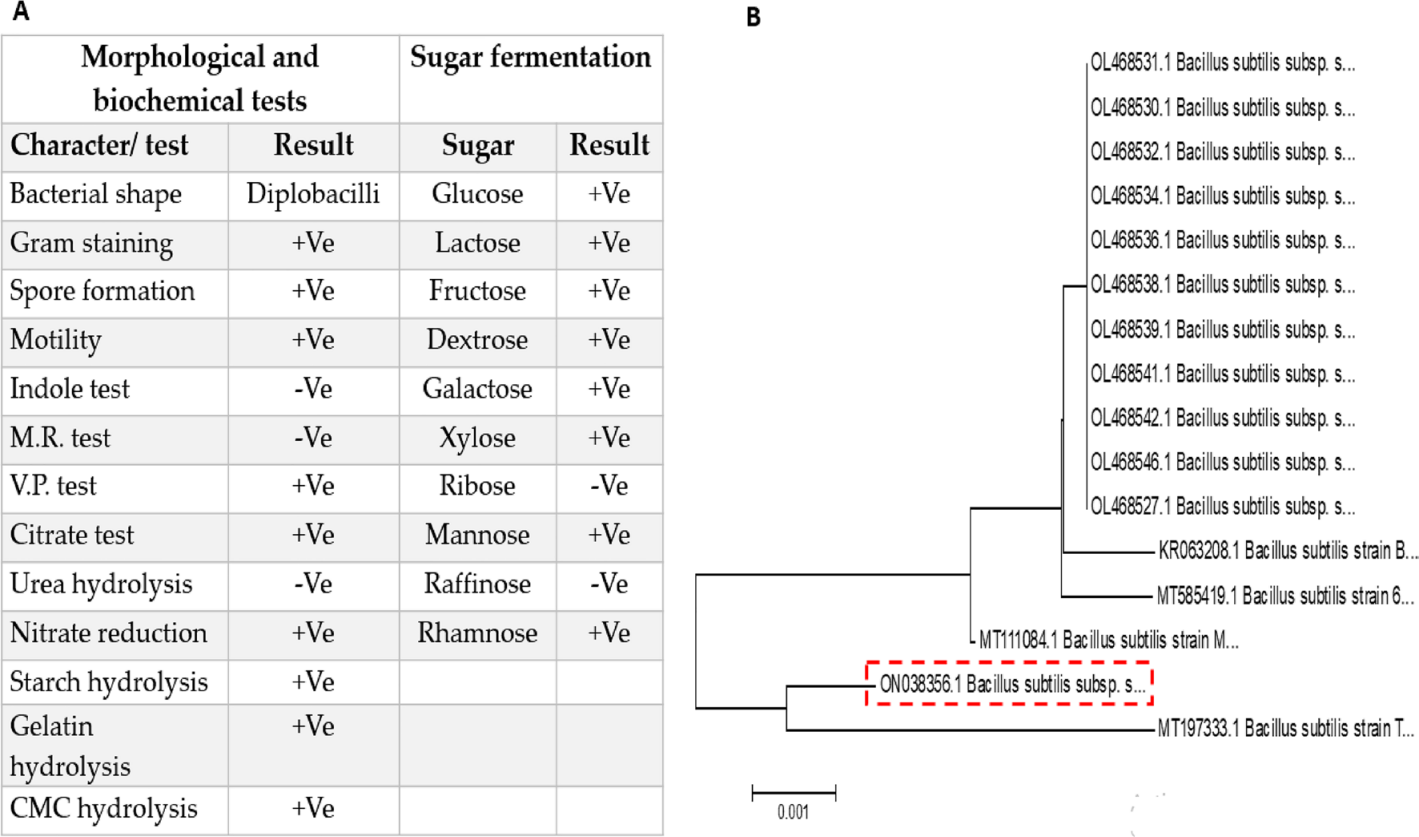
**A** Is the morphological and biochemical characterization of the locally isolated *Bacillus subtilis* ARSE2 where *M.R.* methyl red test and *V.P.* Voges–Proskauer test. **B** is the phylogenetic relationship of the isolate ARSE2 through the neighbor-joining method and based on Jukes–Cantor distances. The phylogenetic analysis was performed by comparing the 16S rRNA sequence for isolated ARSE2 to reference strains using the MEGA 6 software

Additionally, the results (Fig. 3A) indicated that the isolate coded ARSE2 was Gram-positive, motile, spore-forming, facultative aerobic, and rod shape bacillus species. The ARSE2 isolate revealed a significant ability to degrade complex substrates, such as starch, gelatin, and CMC, indicating amylases, proteases, and cellulases activities. Furthermore, the ARSE2 isolate was also able to assimilate a wide range of simple sugars, except ribose and raffinose. At the molecular level, the genomic DNA of the potent xylanases-producing isolate (ARSE2) was isolated and the 16S rRNA gene was amplified. The 16S rRNA sequence was aligned against 16S rRNA sequences of Gen Bank (http://blast.ncbi.nlm.nih.gov/Blast.cgi) through the BLAST algorithm where the gene homology revealed close relation to *Bacillus subtilis* subsp. *subtilis* strain ARSE2, with a similarity of 99% and accession number ON038356.1. The phylogenetic relationship confirmed the close relatedness of the locally isolated ARSE2 to *Bacillus subtilis* species as indicated in Fig. 3B.

### Optimization of xylanase production

#### Effect of different carbon sources on xylanase production

**Fig. 4:**
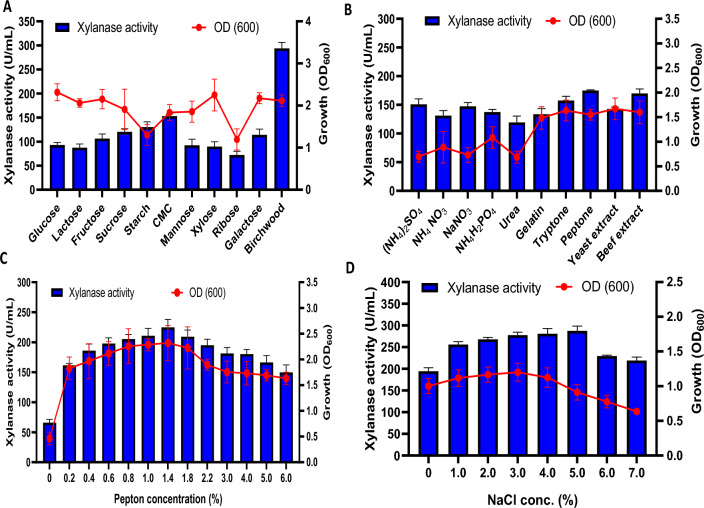
Optimization of the nutritional conditions for maximum xylanase production by *Bacillus subtilis* ARSE2, including the effect of different carbon sources on xylanase production (**A**), the effect of different nitrogen sources on xylanase production (**B**), different concentrations of the optimum nitrogen source (peptone) (**C**), and the effect of different NaCl concentrations on xylanase production

The carbon source is an essential constituent of the fermentation medium, which affects cell growth and metabolic activity. Hence, the effect of various carbon sources on xylanase production by *Bacillus subtilis* ARSE2 was investigated. The results (Fig. 4A) revealed that birchwood xylan supported the highest xylanase production of about 293.86 U/mL (OD_600_ 2.113), followed by CMC and starch with enzyme productivity of 152.87 and 130.09 U/mL, respectively. On the other hand, the other applied carbon sources supported very low xylanase productivity, where the lowest production was about 72 U/mL through ribose. Xylanases are inducible enzymes, largely induced by the soluble catabolites generated from xylan degradation (Gaur et al. 2015; Shakoori et al. 2015). The results indicated growth-independent xylanase production. The highest cell mass (OD_600_ 2.315) was detected from glucose associated with a very low xylanase production (92.92 U/mL), which could be attributed to feedback inhibition (Maulana Hidayatullah et al. 2020).

#### Effect of different nitrogen sources on xylanase production

The nature and concentration of nitrogen sources directly affect cell growth and enzyme production. The results (Fig. 4B) revealed that organic nitrogen sources such as peptone showed the maximum xylanase activity of about 174.93 U/mL, followed by beef extract and tryptone (169.89 and 157.42 U/mL, respectively). Additionally, some inorganic nitrogen sources, such as (NH_4_)_2_SO_4_, NaNO_3_, and NH_4_H_2_PO_4_, supported a significant titer of xylanase of about 150.95, 147.35, and 137.28 U/mL, respectively. These results agree with the other studies that reported an enhancement in xylanase production in *Bacillus* sp. through organic nitrogen sources (Otero et al. 2021; Bakry et al. 2022), which could be attributed to their high content of vitamins and minerals required for better growth and enzyme production. In contrast, other studies found that inorganic nitrogen sources increased xylanase production (Kallel et al. 2016; Pasalari and Homaei 2022). The highest cell mass (OD_600_ 1.669) was supported by yeast extract, but the xylanase production was 142.80 U/mL, which is in line with the previous carbon source results.

Additionally, the effect of different concentrations of peptone on xylanase production by *Bacillus subtilis* ARSE2 is illustrated in Fig. 4C. The xylanase production and stain growth increased with the increase in peptone concentrations, where the highest productivity (224.80 U/mL) and cell growth (OD_600_ 2.320) were at 1.4% peptone. The xylanase production and bacterial growth slightly decreased after this concentration to reach 149.749 U/mL and 1.631 (OD_600_), respectively, at 6% of the peptone concentration.

#### Effect of NaCl concentrations on xylanase production

Bacterial growth and xylanase productivity increased gradually when the concentration of sodium chloride was increased (Fig. 4D). The maximum xylanase production (287.39 U/mL) was at 5% NaCl, while the highest cell mass was at 3% NaCl (OD_600_ 1.201). The optimum NaCl concentration for maximum xylanase production is strain-dependent, as supplementation with 2% NaCl (w/v) showed the highest xylanase production by *Bacillus pumilus* LRF1X (Banerjee and Ghosh 2016), whereas 0.8% NaCl (w/v) was optimum for *Bacillus megaterium* BM07 (Irfan et al. 2016). Both bacterial growth and xylanase production by *Bacillus subtilis* ARSE2 were gradually reduced when the NaCl concentration increased above 5%, which could be attributed to increased osmotic pressure (Wang et al. 2010).

#### Effect of different metals and amino acids on xylanase production

**Fig. 5:**
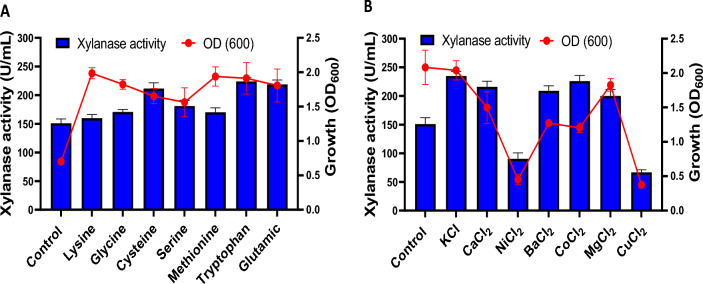
Optimization of medium additives, including** e**ffect of different amino acids (**A**) and metal supplementation (**B**) on xylanase production by *Bacillus subtilis* ARSE2

Metal ions are known to play a crucial role in enzyme activity as cofactors. Hence, the effects of various metal ions (K^+^, Ca^2+^, Ni^2+^, Ba^2+^, Co^2+^, Mg^2+^, and Cu^2+^) on xylanase production were examined as compared to the control (metal-free). The results (Fig. 5A) revealed that xylanase productivity was enhanced in the presence of Ca^2+^, Ba^2+^, and Co^2+^, whereas the maximum enzyme production of 234.88 U/mL was detected by the K^+^ ion compared to the control (203.94 U/mL). These results are in agreement with Tiwari et al. (2022) reported enhancement of xylanase production through the Co^2+^ ion. On the other hand, Ni^2+^, Mg^2+^, and Cu^2+^ reduced the xylanase production to 90.28, 200.11, and 66.78 U/mL, respectively, which is in accordance with the other studies (Lai et al. 2021; Tiwari et al. 2022). This may be due to its interaction with sulfhydryl groups, suggesting that there is an important cysteine residue in or close to the active site of the enzyme (Ajsuvakova et al. 2020). Regarding cell mass production, all applied metal ions exhibited an inhibitory effect on cell growth compared to the control.

Furthermore, the effect of various amino acids on xylanase production was evaluated and is represented in Fig. 5B. Compared to the control, both bacterial growth and xylanase productivity were enhanced with all of the studied amino acids, with maximum enzyme production by tryptophan (223.84 U/mL), followed by glutamic acid (218.57 U/mL), and cysteine (211.62 U/mL). Lysine revealed the lowest xylanase induction potential, about 152.8 U/mL, with approximately no difference from the control.

#### Effect of cultivation temperatures and pH on xylanase production

**Fig. 6:**
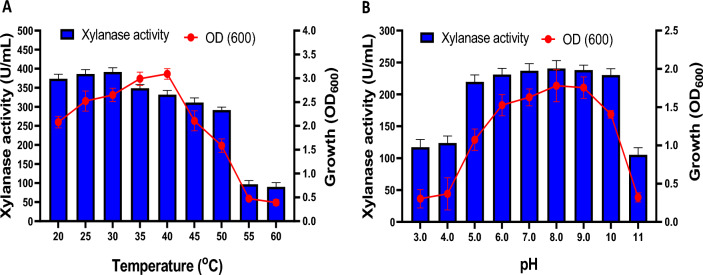
Optimization of physical cultivation conditions, including the effect of different incubation temperatures from 20 to 60 °C (**A**) and different medium pH from 3 to 11 (**B**) on xylanase production and growth of *Bacillus subtilis* ARSE2

The effect of different temperatures (20–60 °C) was studied for maximal xylanase production by *Bacillus subtilis* ARSE2. The results (Fig. 6A) revealed a gradual increase in xylanase production at a temperature range of 20–30 °C, with maximum enzyme production (391.46 U/mL) at 30 °C. Afterward, xylanase productivity gradually decreased with increasing temperature (35–60 °C), with the lowest xylanase activity (89.80 U/mL) at 60 °C. According to the literature, the optimum temperature for xylanase production ranged between 30 and 60 °C, depending on the producing strain (Irfan et al. 2016; Ketsakhon et al. 2022). Furthermore, the bacterial growth was gradually increased with temperature and maximized at 40 °C (OD_600_ 3.091). At temperatures above 40 °C, the growth rate abruptly decreased.

On the other hand, medium pH significantly affected various enzymatic processes and nutrient transportation through the cell membrane (Kapoor et al. 2008). Hence, the effect of different pH values on xylanase production by *Bacillus subtilis* ARSE2 was evaluated, as represented in Fig. 6B. The results revealed significant xylanase production in a slightly acidic-to-alkaline medium at a pH range of 5–10. The maximum enzyme production was at pH 8 (240.63 U/mL), which is consistent with the other studies (Irfan et al. 2016; Danso et al. 2022). The enzyme productivity was dramatically retarded in extremely acidic (pH 3–4) and hyperalkaline (pH 11) media, which could be related to enzyme denaturation. On the other hand, bacterial growth significantly increased from pH 3 to 9, with maximum growth at pH 8.

#### Effect of inoculum size and incubation period on xylanase production

**Fig. 7:**
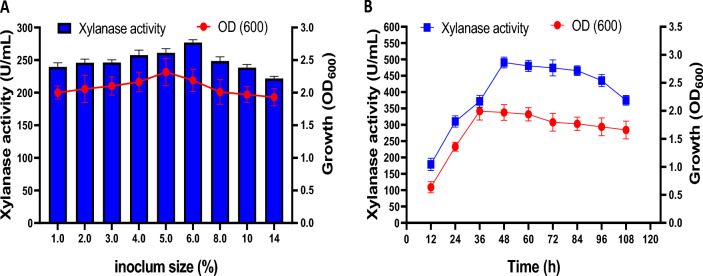
Effect of different inoculation volume (**A**) and incubation time (**B**) on bacterial growth and xylanase production by *Bacillus subtilis* ARSE2

The effect of different inoculum sizes of *Bacillus subtilis* ARSE2 upon xylanase production was evaluated. The results (Fig. 7A) revealed that the optimum inoculum size was 6%, with a maximum enzyme productivity of about 276.84 U/mL. Lower or higher inoculum sizes (more or less 6%) reduced xylanase production, which could be attributed to an insufficient number of bacteria or rapid depletion of nutrients in the fermentation medium (Battan et al. 2007; Omojasola et al. 2008).

The optimum cultivation time for maximum enzyme production is likely to depend on several factors, including the strain type and cultivation requirements (Danso et al. 2022). In the present study (Fig. 7B), xylanase production started during the log phase, and its levels increased gradually during the fermentation course, with maximum xylanase production occurring at 48 h (about 490 U/mL). Prolonged incubation decreased enzyme activity (about 374.8 U/mL after 108 h), which could be attributed to the proteolysis of the enzyme and/or the production of toxic metabolites (Maulana Hidayatullah et al. 2020). These results are in agreement with the other studies that reported maximum xylanase production at 48 h from several *Bacillus* sp. (Irfan et al. 2016; Malhotra and Chapadgaonkar 2021). On the other hand, the bacterial growth increased over time to a maximum (OD_600_ 1.994) after 36 h. The cells maintained a stationary growth between 36 and 60 h of fermentation and then declined.

#### Application of lignocellulosic wastes for sustainable xylanase production

Commercial xylanase production implies a reduction in the medium cost and operation condition requirements (Bajar et al. 2020). The effect of alkali-pre-treated agro-industrial wastes on xylanase production by* Bacillus subtilis* ARSE2 through SSF or SF fermentation approaches was investigated. The results of SSF (Table 1) revealed that all tested wastes supported xylanase production at different titers. The maximum xylanase production was through sugarcane bagasse (472.03 U/mL) under SSF conditions. Accordingly, enhanced xylanase production employing multiple agricultural substrates, as well as high levels of enzyme production at lower substrate-to-moisture ratios, have previously been reported (Ketsakhon et al. 2022; Kaur et al. 2023). Sugarcane bagasse is an inexpensive byproduct with high xylan content; therefore, it is one of the most utilizable components of media for xylanase production (Alokika and Singh 2020; Moran-Aguilar et al. 2021). The other wastes also supported varied xylanase production, where sawdust reported the minimum xylanase production (243.03 U/mL).

**Table 1:** Xylanase production under solid-state (SSF) and submerged fermentation (SF) using different lignocellulosic wastes

Agro-industrial wastes	Xylanase activity (U/mL)	
	SSF (16%)	SF (2%)
Wheat straw	416.88 ± 11.1	457.40 ± 11.4
Rice straw	454.28 ± 13.4	458.12 ± 17.6
Sugarcane bagasse	472.03 ± 8.9	464.83 ± 15.3
Corn stalks	438.70 ± 11.2	429.82 ± 6.8
Woody sawdust	243.03 ± 7.9	485.70 ± 4.8
Cotton stalks	316.88 ± 14.8	404.17 ± 14.3
Bean straw	458.36 ± 6.93	441.81 ± 10.5

On the other hand, the SF results revealed that sawdust was found to be the most suitable substrate for maximum xylanase production by *Bacillus subtilis* ARSE2, followed by sugarcane bagasse, with activities of 485.70 and 464.83 U/mL, respectively. Currently, the implementation of pre-treated agricultural residues is widely reported for sustainable xylanase production, including rice straw, wheat straw, sugarcane bagasse, wheat bran, oat husk, rice husk, rice bran, and oat wheat (Bajar et al. 2020; Shakir et al. 2020; Danso et al. 2022).

### Purification of the xylanase enzyme produced by Bacillus subtilis ARSE2

**Fig. 8:**
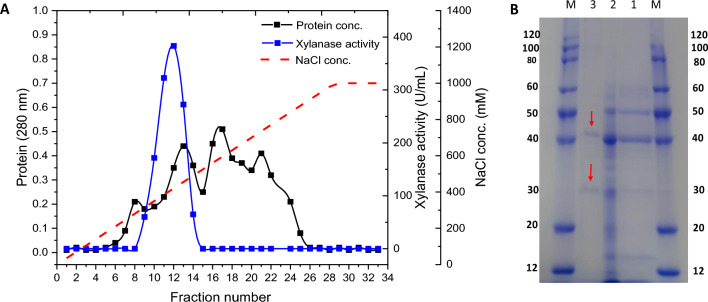
Purification chromatogram of xylanase on DEAE column (**A**) revealing protein concentration and xylanase activity (U/mL) as eluted with NaCl gradient (mM), whereas (**B**) is the SDS-PAGE representing the purification progress, including lane M: protein marker, lane 1: crude enzyme, lane 2: ammonium sulfate precipitation, and lane 3: DEAE-column results

For xylanase purification, the CFS (150 mL) was concentrated by (NH_4_)_2_SO_4_ (75% saturation) and applied to the DEAE column. As indicated in the purification results (Table 2), the protein content in the CFS (2750.7 mg) was decreased to 91.5 mg upon (NH_4_)_2_SO_4_ precipitation and dialyses. Application of (NH_4_)_2_SO_4_ increased the xylanase purity 7.5-fold compared to the crude enzyme. The DEAE-column chromatogram (Fig. 8A) revealed several protein peaks in the applied dialyzed sample, where xylanase activity was detected at fractions 9–14, with maximum activity at fractions 10–13. The DEAE-column application enhanced the specific activity of xylanase to 348.8 U/mg compared to CFS (27.95 U/mg), which indicated a significant reduction in untargeted protein. The results indicated a 12.5-fold increase in the xylanase protein purity compared to CFS (Table 2). The SDS-PAGE (12%) results confirmed the purification progress, where two protein bands were detected after DEAE-column application at 42 and 30 KDa (Fig. 8B).

**Table 2:** The purification steps of the xylanase enzyme represent total protein and activity related to total sample volume, specific activity (total activity/total protein), yield (total activity at each step/CFS total activity $\times 100$), and purification fold (specific activity at each step/CFS specific activity)

Purification steps	Volume (mL)	Total protein (mg)	Total activity (U)	Specific activity (U/mg)	Yield (%)	Purification (fold)
Cell-free supernatant (CFS)	150	2750.7	76,882.5	27.95	100	1
Ammonium sulfate	15	91.6	19,220.6	210.06	25	7.5
DEAE column	3	12.5	4360	348.8	5.7	12.5

### Characterization of the partially purified xylanase

#### Effect of temperatures on xylanase activity and stability

**Fig. 9:**
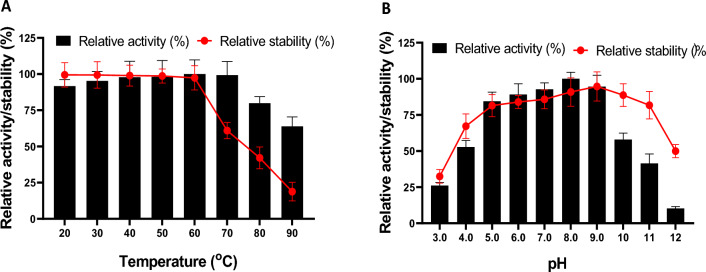
Effect of different temperatures (**A**) and pH (**B**) on both activity and stability of partially purified xylanase enzyme

The optimum temperature for partially purified xylanase activity was evaluated at 20–90 °C. As shown in Fig. 9A, the xylanase enzyme was active over a wide temperature range. The maximum enzyme activity was at 60–70 °C (about 980.14 U/mL), which is in agreement with the other studies that reported the same temperature for optimum xylanase activity from several *Bacillus* sp. (Dheeran et al. 2012; Saleem et al. 2012)*.* Increasing the reaction temperatures over 70 °C gradually reduced the xylanase activity, with a minimum activity at 90 °C of about 625.73 U/mL.

Additionally, the thermal stability study (Fig. 9A) revealed the high stability of the purified xylanase, as indicated by retaining over 97.4% of its hydrolytic activity after 60 min of incubation at a temperature of 20–60 °C. Temperatures above 60 °C decreased the enzyme stability significantly, with more than 55- and 80% of enzyme activity loss at 80- and 90 °C, respectively. The thermal stability of *Bacillus subtilis* ARSE2 xylanase in the current study is comparable to that of *Thermobifida halotolerans* YIM 90462 (Zhang et al. 2012) and *Geobacillus* sp. WBI xylanases, which revealed 0% relative activity after 30–60 min at 80 °C (Mitra et al. 2015).

#### Effect of different pH values on xylanase activity and stability

The effect of pH on xylanase activity was evaluated at different pH values (3–12). As shown in Fig. 9B, the studied xylanase was active in a wide pH range (acidic–alkaline), with more than 41% of its activity at a pH between 4.0 and 11.0. The optimum pH value for xylanase activity was 8.0, which is in line with several studies (Patel and Dudhagara 2020b; Shakir et al. 2020). However, other pH values for optimum xylanase activity were also reported (Bakry et al. 2022; Tiwari et al. 2022). The enzyme activity was negatively affected by pH values less than 5 or greater than 9.

The stability of partially purified xylanase at the previous pH values was also evaluated. The results (Fig. 9B) indicated that the partially purified xylanase revealed significant stability in the pH range 4–11 (about 67.23% and 81.7%, respectively), which is in line with optimum pH value results. The maximum enzyme stability was at pH 8–9 (about 95%), which indicated the suitability of isolate enzymes for pulping and food industries that carried on at high pH values (Kumar et al. 2014; Lai et al. 2021). These results are in agreement with those reported by Chivero et al.**,** who reported that xylanase from the genus *Bacillus* has an optimum pH of 8.0 and is stable in a wide pH range of 6.0–9.0 (Chivero et al. 2001)**.**

#### Effect of different metal ions on the activity and stability of xylanase

Inhibition of xylanase activity through metal ions accumulated in woody and agricultural residues is a major challenge for enzyme application in the pulping process (Poornima et al. 2020). Hence, the effects of Ba^2+^, Mg^2+^, Ca^2+^, Ni^2+^, Co^2+^, and Mn^2+^ at different concentrations on the activity and stability of partially purified xylanase were evaluated and are presented in Table 3. The xylanase activity and stability were slightly enhanced with Ba^2^ and Co^2+^ ions compared to the control. At 1 mM concentration, the Ba^2^ and Co^2+^ ions enhanced both xylanase activity and stability to 101.5 and 111.69%, respectively (with Ba^2^) and 116.4 and 129.36%, respectively (with Co^2+^). Furthermore, Mg^2+^ and Mn^2+^ ions enhanced the xylanase activity, but the enzyme stability was adversely affected. The maximum enzyme activity was 137% at 5 mM of MgCl_2_, whereas the enzyme stability decreased to 82.4% when incubated for 1 h with Mg^2+^ at 10 mM. Also, Mn^2+^ ions enhanced the enzyme activity to 111.15 at 1 mM, with a maximum reduction in the enzyme stability (about 30%) at 10 mM compared to the control. The results are in accordance with several studies that have reported the enhancement of xylanase activity with Mn^2+^ ions (Bataillon et al. 2000; Kiddinamoorthy et al. 2008). The Ca^2+^ ions revealed no significant effects on both xylanase activity and stability, as the maximum enzyme activity was 101.95% (at 1 mM), while the minimum stability (99.61%) was at 10 mM. Among the studied metals, the Ni^2+^ ions moderately affected the xylanase activity and stability by 76.5% and 93.3%, respectively, at a concentration of 10 mM.

**Table 3:** Effect of different metal ions (1–10 mM), detergents (1–10%), and some enzyme inhibitors (1–10 mM) on the activity and stability of the partially purified xylanase

Metal ions				Detergent and enzyme inhibitors			
Name	Conc	Relative activity (%)	Relative stability (%)	Name	Conc	Relative activity (%)	Relative stability (%)
Control	0	100 ± 0.0	100 ± 0.0	Control	0	100 ± 0.0	100 ± 0.0
BaCl_2_ (mM)	1	101.5 ± 7.8	111.7 ± 5.1	Tween-80 (%)	1	120.3 ± 1.8	115.8 ± 2.4
	5	100.6 ± 6.5	108.9 ± 3.2		5	124.7 ± 2.7	116.8 ± 3.1
	10	100.1 ± 2.3	106.4 ± 2.1		10	126.1 ± 4.3	117.5 ± 7.2
MgCl_2_ (mM)	1	111.8 ± 4.7	87.6 ± 4.3	Urea (%)	1	94.8 ± 1.3	93.0 ± 1.8
	5	103.7 ± 1.2	85.4 ± 2.7		5	94.6 ± 4.3	89.0 ± 3.4
	10	96.9 ± 1.7.0	82.4 ± 1.7		10	92.8 ± 4.2	84.2 ± 2.5
CaCl_2_ (mM)	1	102.0 ± 3.2	109.8 ± 6.4	H_2_O_2_ (%)	1	97.5 ± 3.1	86.4 ± 2.1
	5	100.4 ± 4.3	103.7 ± 2.7		5	00 ± 0.0	00 ± 0.0
	10	99.8 ± 7.2	99.6 ± 1.7		10	00 ± 0.0	00 ± 0.0
NiCl_2_ (mM)	1	95.3 ± 4.0	98.1 ± 5.4	DTT (mM)	1	105.0 ± 1.7	105.4 ± 3.0
	5	79.2 ± 2.3	96.8 ± 9.8		5	105.8 ± 1.8	110.7 ± 2.1
	10	76.5 ± 1.7	93.3 ± 3.7		10	108.0 ± 4.1	110.7 ± 4.0
CoCl_2_ (mM)	1	116.4 ± 2.7	129.4 ± 9.4	EDTA (mM)	1	97.0 ± 3.6	97.0 ± 4.2
	5	115.6 ± 1.9	114.9 ± 8.7		5	87.0 ± 2.3	82.5 ± 1.5
	10	115.6 ± 1.8	111.4 ± 6.2		10	80.9 ± 4.2	78.1 ± 4.2
MnCl_2_ (mM)	1	111.2 ± 4.7	79.7 ± 2.3	SDS (mM)	1	99.7 ± 6.2	15.0 ± 1.1
	5	107.9 ± 01.7	76.4 ± 2.7		5	51.1 ± 2.4	14.2 ± 0.9
	10	106.1 ± 3.6	69.5 ± 3.4		10	30.9 ± 2.0	13.9 ± 1.3

#### Effect of some detergents and enzyme inhibitors on the activity and stability of the partially purified xylanase

The effects of some detergents and enzyme inhibitors on xylanase activity and stability were evaluated at different concentrations. As shown in Table 3, xylanase activity and stability were enhanced through tween-80 and DTT. Tween-80 supported the maximum enzyme activity and stability of about 126.1% and 117.5%, respectively, followed by DTT (108% and 110.7%, respectively), at the same concentration (10%). The results are in line with several previous studies (Park et al. 2012; Wang et al. 2022). The enhancement in xylanase activity and/or stability through DTT could be related to its role in stabilizing the conformational folding of the enzyme by prohibiting disulfide bond formation at the irregular position of the protein (Vieira et al. 2007).

On the other hand, SDS, EDTA, urea, and H_2_O_2_ adversely affected both enzyme activity and stability at all applied concentrations. H_2_O_2_ severely affected xylanase activity and stability, with complete enzyme inhibition at concentrations greater than 1%. The SDS negative effect comes next to H_2_O_2_, where xylanase activity and stability were reduced to about 30% and 14%, respectively, at 10 mM SDS. The xylanase stability toward SDS in the current study (99.7% at 1% SDS) is comparable to that of *Geobacillus thermodenitrificans* xylanase, which retains 72% activity at 0.5% SDS (Verma et al. 2013), and to *Paenibacillus* sp. NF1 (Zheng et al. 2014), which is significantly inhibited even at 0.02% SDS. In the same regard, the increase in EDTA concentration reduced both xylanase activity and stability to 80.9% and 78.1%, respectively, at a 10 mM concentration. The inhibition of *Bacillus subtilis* ARSE2 xylanase activity in the presence of EDTA suggests that metals are desirable for the optimum enzymatic reaction, confirming the metalloprotein nature of the enzyme (Khandeparkar and Bhosle 2006), which is in line with the enhancement in the xylanase activity in the presence of CoCl_2_ and MgCl_2_ (Table 3). Furthermore, the inhibition of xylanase activity through EDTA has been widely reported (Amobonye et al. 2021; Olopoda et al. 2022).

#### Effect of substrate concentrations on xylanase activity and kinetics

**Fig. 10:**
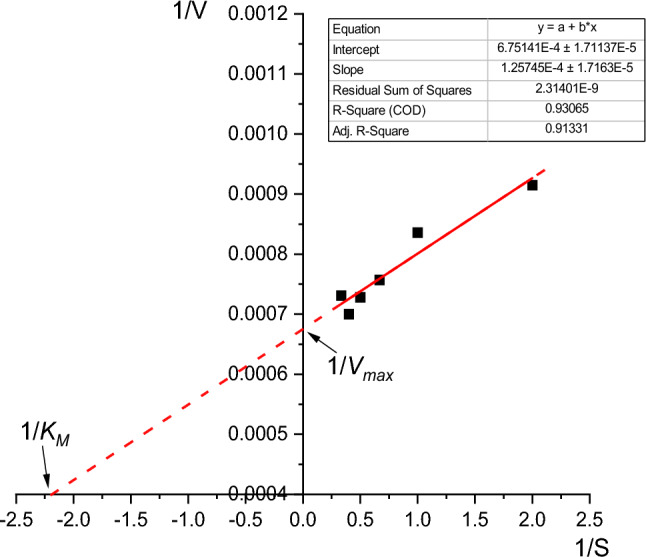
The relation between birchwood xylan concentration (S) and xylanase velocity (V) as represented through the Lineweaver–Burk plot by plotting 1/substrate concentration (1/S) *vs*. 1/enzyme velocity (1/V)

The effect of different concentrations of birchwood xylan (0.5–5.0%) on partially purified xylanase activity was studied. The results (Fig. 10) showed that xylanase activity increased gradually as substrate concentration increased from 0.5 to 2%, with maximum xylanase activity (1429.03 U/mL) at 2.5% of the birchwood xylan concentration. Afterward, the increase in the birchwood xylan concentration insignificantly affected the enzyme activity, indicating a substrate saturation state. The Lineweaver–Burk plot (Fig. 10) represented the relationship between enzyme velocity and substrate concentration, where *K*_*m*_ and *V*_*max*_ values could be deduced based on the Michaelis–Menten kinetics model. The results indicated that *V*_*max*_ was 1481.5 U/mL when the *K*_*m*_ constant was 0.187 mM. The very low *K*_*m*_ value (0.187 mM) indicated the high affinity and efficiency of xylanase from *Bacillus subtilis* ARSE2 to degrade xylan complex polysaccharide (Yadav et al. 2018; Tiwari et al. 2022).

### Evaluation of XOS antimicrobial activity

**Fig. 11:**
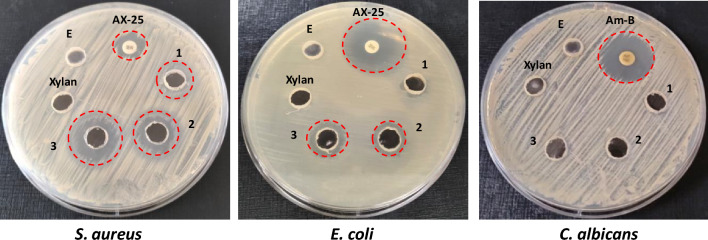
The antimicrobial activity of the XOS prepared through purified xylanase at three different hydrolysis times: 15 min (1), 30 min (2), and 60 min (3). Non-hydrolyzed xylan (Xylan) and purified xylanase (E) were also included. Two antimicrobial drugs, amoxicillin (AX-25) and amphotericin-B (Am-B), were included for bacteria and fungi, respectively

The worldwide spread of multidrug-resistant bacteria has been a pressing human health challenge in recent decades (Thoma et al. 2022). Increased research toward novel antimicrobial drugs with lower resistant induction potential is mandatory (Murray et al. 2022). Hence, the antimicrobial activity of the prepared XOS at three hydrolysis times (15, 30, and 60 min) was evaluated against three human pathogens. The results (Fig. 11) indicated broad-spectrum antibacterial activity for the prepared XOS against *S. aureus* and *E. coli.* However, no antifungal activity was detected against* C. albicans.* The antibacterial activity was more significant for the applied Gram-positive (*S. aureus*) bacterium compared to the Gram-negative one (*E. coli*), which is in accordance with the other study (Christakopoulos et al. 2003)*.* Additionally, the antibacterial activity was hydrolysis time-dependent, as indicated in Table 4. Increasing the hydrolysis time from 15 to 60 min increased the antimicrobial activity from 15.1 ± 1.2 to 20.7 ± 1.5 mm and 00 ± 00 to 13 ± 0.89 mm toward *S. aureus* and* E. coli*, respectively. Collectively, the results indicated that the potency of prepared XOS against *S. aureus* with antibacterial activity was higher than that of the amoxicillin reference drug (12.5 ± 0.98 mm). Sun et al. attributed this inhibition to the ability of XOS to interfere with and downregulate the pathogenicity and quorum-sensing genes in *S. aureus *ATCC 6538 (Sun et al. 2022). However, targeting the bacterial cell wall structure could be a part of this activity, which may be supported by higher activity in Gram-positive and the complete absence of antifungal activity toward *C. albicans* in the current study.

**Table 4:** The antimicrobial activity of the prepared XOS expressed with halo-zone diameter in millimeters (mm) at three hydrolysis times of 15, 30, and 60 min

Organism	Positive controls		Xylan hydrolysis time (min)			Negative controls	
	AX-25	Am-B	15	30	60	Xylan	E
*S. aureus*	12.5 ± 0.98	–	15.1 ± 1.2	18.5 ± 1.7	20.7 ± 1.5	00 ± 00	00 ± 00
*E. coli*	26 ± 1.25	–	00 ± 00	11 ± 0.71	13 ± 0.89	00 ± 00	00 ± 00
*C. albicans*	–	21 ± 2.25	00 ± 00	00 ± 00	00 ± 00	00 ± 00	00 ± 00

## Conclusion

The increased worldwide demand for the efficient valorization of lignocellulosic materials for value-added products forces the continuous search for efficient lignocellulose-degrading enzymes with promising characteristics. In the current study, a local strain (ASRE2) belonging to *Bacillus subtilis* revealed the highest xylanase titer (212.7 U/mL). The cultivation conditions directly influence the xylanase production, with maximum xylanase production (490.8 U/mL) at optimum conditions representing a 2.2-fold increase compared to the basal medium xylanase level (221.69 U/mL). Exploiting the lignocellulosic wastes for xylanase production reveals that the fermentation type directly influences the waste type and concentration for maximum enzyme production. Under solid-state fermentation, sugarcane bagasse (16% conc.) supported the maximum enzyme (472.03 U/mL), whereas woody sawdust had the highest enzyme titer of 485.70 under submerged fermentation. The produced enzyme was partially purified through the DEAE column to about 12.5 purification fold, where the purified enzyme revealed strong thermal and pH stability with remarkable stability toward several detergents and enzyme inhibitors, which encourages its implementation in several industrial applications. Additionally, the XOS resulting from xylan hydrolysis with the purified enzyme revealed significant broad-spectrum antibacterial activity, especially toward *S. aureus,* which paves the way for intensifying the research in this direction toward novel antimicrobial agents. The current study asserts the applicability of xylanases from locally isolated *Bacillus subtilis* for industrial applications and the production of biologically active XOS. However, further research is required to determine the enzyme's exact molecular weight and identify the nature of biologically active XOS and their mechanisms of action.

## Data Availability

All datasets used/or analyzed during the current study are available from the corresponding authors on reasonable request.
